# O-GlcNAc transferase regulates intervertebral disc degeneration by targeting FAM134B-mediated ER-phagy

**DOI:** 10.1038/s12276-022-00844-7

**Published:** 2022-09-02

**Authors:** Rongjin Luo, Gaocai Li, Weifei Zhang, Huaizhen Liang, Saideng Lu, Jason Pui Yin Cheung, Teng Zhang, Ji Tu, Hui Liu, Zhiwei Liao, Wencan Ke, Bingjin Wang, Yu Song, Cao Yang

**Affiliations:** 1 Department of Orthopaedics, Union Hospital, Tongji Medical College, Huazhong University of Science and Technology, Wuhan, 430022 China; 2 Department of Spine Surgery, Honghui Hospital, Xi’an Jiaotong University, Xi’an, 710054 China; 3 Department of Orthopaedics and Traumatology, The University of Hong Kong, Hong Kong SAR, 000000 China; 4 Spine Labs, St. George and Sutherland Clinical School, University of New South Wales, Kogarah, NSW 2217 Australia

**Keywords:** Autophagy, Glycosylation, Apoptosis, Mechanisms of disease, Senescence

## Abstract

Both O-linked β-N-acetylglucosaminylation (O-GlcNAcylation) and endoplasmic reticulum-phagy (ER-phagy) are well-characterized conserved adaptive regulatory mechanisms that maintain cellular homeostasis and function in response to various stress conditions. Abnormalities in O-GlcNAcylation and ER-phagy have been documented in a wide variety of human pathologies. However, whether O-GlcNAcylation or ER-phagy is involved in the pathogenesis of intervertebral disc degeneration (IDD) is largely unknown. In this study, we investigated the function of O-GlcNAcylation and ER-phagy and the related underlying mechanisms in IDD. We found that the expression profiles of O-GlcNAcylation and O-GlcNAc transferase (OGT) were notably increased in degenerated NP tissues and nutrient-deprived nucleus pulposus (NP) cells. By modulating the O-GlcNAc level through genetic manipulation and specific pharmacological intervention, we revealed that increasing O-GlcNAcylation abundance substantially enhanced cell function and facilitated cell survival under nutrient deprivation (ND) conditions. Moreover, FAM134B-mediated ER-phagy activation was regulated by O-GlcNAcylation, and suppression of ER-phagy by FAM134B knockdown considerably counteracted the protective effects of amplified O-GlcNAcylation. Mechanistically, FAM134B was determined to be a potential target of OGT, and O-GlcNAcylation of FAM134B notably reduced FAM134B ubiquitination-mediated degradation. Correspondingly, the protection conferred by modulating O-GlcNAcylation homeostasis was verified in a rat IDD model. Our data demonstrated that OGT directly associates with and stabilizes FAM134B and subsequently enhances FAM134B-mediated ER-phagy to enhance the adaptive capability of cells in response to nutrient deficiency. These findings may provide a new option for O-GlcNAcylation-based therapeutics in IDD prevention.

## Back pain: Preserving the spine’s padding

A cellular ‘housekeeping’ mechanism that counters the detrimental effects of stress could also help protect against lower back pain by preventing degeneration of the spongy discs that cushion our vertebrae. When subjected to traumatic conditions such as nutrient deprivation, some cells respond by breaking down excess components of an intracellular organelle, the endoplasmic reticulum (ER). Researchers led by Yu Song and Cao Yang at Huazhong University of Science and Technology, Wuhan, China, have shown that this ‘ER-phagy’ response helps promote the survival of stressed nucleus pulposus (NP) cells, the inner core of intravertebral discs. Cultured human NP cells tend to die off in starvation conditions, but were sustained by activation of ER-phagy pathways. This same mechanism was shown to prevent disc degeneration in rats, suggesting a potential therapeutic strategy for preventing lower back pain in humans.

## Introduction

More than 85% of the general population worldwide is afflicted with low back pain at some point throughout their lifetime, in some cases, resulting in disability and presenting a major challenge to global health systems^1,2^. This negative impact on society is increasing with the increase in the aging population. Intervertebral disc (IVD) degeneration (IDD) is widely recognized as the leading cause of low back pain. The etiology and pathophysiology of IDD are complicated. Diverse risk factors, including genetic factors, aging, mechanical stress, obesity, and nutrient availability, have been implicated in the initiation and progression of IDD^3–5^. Nucleus pulposus (NP) cells, the most basic functional unit resident in NP tissue, are critical for the anabolic and catabolic balance of extracellular matrix components such as proteoglycans and collagen II to maintain the gelatinous properties of the NP. Abnormal NP cell apoptosis and impaired cellular activity accompanying aging have been confirmed to be closely associated with IDD^6^. In addition, an increasing number of studies have reported that interventions targeting NP cell apoptosis efficiently alleviated IDD in vitro and in animal studies^7–9^. Therefore, elucidating the mechanisms underlying NP cell apoptosis and the decline in cellular activity may increase the understanding of IDD pathogenesis and lead to potential therapeutic strategies.

The endoplasmic reticulum (ER) is a specialized organelle that consists of extensively organized tubular and lamellar membrane structures; it is highly dynamic and maintains constant turnover in response to various biological stimuli. The ER is a crucial site of protein synthesis, folding, assembly, trafficking, and degradation, including proteins destined for organelles and the extracellular space^10^. Notably, recent studies have indicated that in addition to well-defined ER quality and quantity control mechanisms such as ER-associated protein degradation (ERAD), ER-phagy (namely, reticulophagy) can restore ER homeostasis by selectively delivering excessive ER membrane fragments and ER-resident proteins to lysosomes for degradation under deleterious conditions such as nutrient deprivation, inflammation, oxidative stress, and hypoxia^11,12^. The high selectivity for ER fragments engulfed by the double-membrane autophagosome is largely dependent on ER-phagy adaptors that reside in ER membranes and contain an LC3-interacting region (LIR). Recently, several ER-resident proteins have been identified as ER-phagy adaptors in mammalian cells; these adaptors include FAM134B (family with sequence similarity 134, member B), SEC62 (SEC62 homolog), RTN3L (reticulon 3 long isoform), CCPG1 (cell-cycle progression gene 1), ATL3 (atlastin 3), and TEX264 (testis-expressed 264)^13–18^. Among these proteins, FAM134B was the first identified ER-phagy mediator, and it can restore ER homeostasis by promoting membrane remodeling, ER scission, and targeting ER fragments for lysosomal degradation^13^. Moreover, recent studies have demonstrated that ER-phagy dysfunction plays a pivotal role in the pathogenesis of multiple human disorders, including cancers, viral infections, and neurodegenerative diseases^19–21^.

O-linked β-N-acetylglucosaminylation (O-GlcNAcylation) is a ubiquitous, dynamic, and reversible posttranslational modification in which a single beta-N-acetylglucosamine (O-GlcNAc) moiety is covalently attached to serine/threonine residues of myriad nucleocytoplasmic proteins, such as receptors, kinases, cytoskeletal proteins, and transcription factors. Emerging evidence suggests that O-GlcNAcylation plays fundamental roles in numerous physiological processes, including gene transcription, signal transduction, energy metabolism, and cell proliferation and death, by altering protein stability, interactions, subcellular localization, and function^22,23^. Interestingly, in contrast to most other posttranslational modifications, O-GlcNAcylation is orchestrated by only two conserved enzymes, namely, O-GlcNAc transferase (OGT) and O-GlcNAcase (OGA), which are critical for catalyzing the addition and removal of O-GlcNAc, respectively^24^. Physiologically, O-GlcNAcylation is optimally maintained in a dynamic balance through the mutual regulation of OGT and OGA, and disruption of O-GlcNAc homeostasis has been documented to be associated with the pathogenesis of various human diseases^24,25^. O-GlcNAc cycling has been validated to be closely correlated with the regulation of multiple steps of autophagy, including phagophore initiation, elongation, and ultimately fusion with lysosomes^26–28^. In addition, enhanced O-GlcNAcylation and OGA/OGT expression have been observed in human degenerated intervertebral disc tissues^29^. However, the related functional roles and underlying mechanisms of IDD remain largely unknown. Thus, research is actively focusing on O-GlcNAcylation in IDD.

As both O-GlcNAcylation and FAM134B-mediated ER-phagy are highly sensitive to stress conditions, especially nutrient deprivation, we further evaluated their association in IDD. We found elevated OGT expression and O-GlcNAcylation in degenerated NP tissues and human NP cells under nutrient deprivation (ND) conditions. Further investigation revealed that the extent of FAM134B-mediated ER-phagy was modulated by O-GlcNAcylation under ND conditions. Mechanistically, our data demonstrated that OGT directly interacted with the FAM134B protein, and increased FAM134B stability by inhibiting its ubiquitin-mediated degradation. These novel findings may contribute to understanding the connections among O-GlcNAcylation, FAM134B-mediated ER-phagy, and NP cell damage in the pathophysiology of IDD and provide new insights into the development of potential therapeutic strategies for IDD.

## Materials and methods

### Ethics approval and consent to participate

All experimental procedures involving NP tissue collection and use in research were approved by the Ethics Committee of Tongji Medical College, Huazhong University of Science and Technology (No. S341), and written informed consent was obtained from all participants or their guardians before tissue acquisition. All animal experiments were approved by the Animal Experimentation Committee of Huazhong University of Science and Technology and performed in accordance with ethical regulations (S2394).

### NP tissue collection, cell culture, and treatment

Healthy control NP tissues were obtained from six patients with adolescent idiopathic scoliosis (three males and three females; average age, 15.5 years; range, 13–21 years) who underwent deformity correction surgery. Degenerated NP tissues were acquired from six patients with lumbar spinal stenosis (three males and three females; average age, 55.2 years; range, 43–63 years) undergoing disc excision and spinal fusion surgery. Correspondingly, the IDD grade of each segment was determined with preoperative sagittal T2-weighted magnetic resonance imaging (MRI) according to the Pfirrmann classification systems^30^. Details on the patient demographic characteristics are listed in Supplementary Table 1. For cell isolation and culture, freshly collected NP tissues from adolescent scoliosis donors obtained during surgery were washed and cut into small fragments, and 0.2% collagenase type II (Invitrogen, Carlsbad, CA, USA) was used to digest the tissue at 37 °C for 6 h. Then, the tissue suspension was centrifuged at 1000 × *g* for 5 min, and the collected cells were resuspended in DMEM/F12 supplemented with 10% FBS and 1% penicillin/streptomycin at 37 °C in 5% CO_2_. NP cell characteristics were confirmed as described in our previous study^31^. NP cells at passages 2 and 3 were used in subsequent experiments.

For in vitro experiments, NP cells were incubated with glucose-free DMEM/F12 without FBS supplementation (nutrient deprivation, ND) for different durations (0, 6, 12, 24, and 36 h) or cotreated with ND and 10 μM TMG or 25 μM OSMI-1 for 36 h. For experiments involving transient lentiviral transduction and siRNA transfection, human NP cells were transduced with targeted lentiviral vectors for 72 h or with siRNA for 48 h, followed by ND for 36 h. For the CHX chase assay, NP cells were incubated with CHX (50 μg/mL) for the indicated time period after incubation with TMG or OSMI-1 for 24 h and were then harvested.

Information on reagents and antibodies used in this study were listed in Supplementary Table 2.

### Lentiviral transduction and siRNA transfection

Lentiviral vectors expressing HA-tagged OGT (NM_181672) in the Ubi-MCS-3FLAG-SV40-puromycin plasmid and FLAG-tagged FAM134B (NM_001034850) in the Ubi-MCS-SV40-neomycin plasmid were designed and constructed by GeneChem (Shanghai, China) following standard protocols. For viral transduction, human NP cells were seeded, and after reaching 40–60% confluence, were infected with lentivirus at a multiplicity of infection (MOI) of 50. After 12 h of transduction, the culture medium was changed every other day. When the transduced NP cells were confluent, they were passaged for further experiments. For RNA interference, OGT siRNAs with the following sequences were purchased from RiboBio Co., Ltd. (Guangzhou, China): *OGT* siRNA1, 5′TGAGCAGTATTCCGAGAAA3′, and *OGT* siRNA2, 5′CAATCATTTCATTGATCTT3′. In addition, *FAM134B* siRNA (5′AGCTATCAAAGACCAGTTA3′) and *OGA* siRNA (5′GAAATCTATCAGTACCTAGGA3′) were obtained from Qijing Biotechnology Co. (Wuhan, China). siRNAs were transfected using Lipofectamine 2000 (Invitrogen) following the manufacturer’s instructions. Transfection efficiency was evaluated through western blot analysis.

### SA-β-gal staining

Senescence‐associated β‐galactosidase (SA‐β‐gal) staining of human NP cells was performed utilizing a senescence-associated β-galactosidase staining kit (Beyotime) according to the manufacturer’s instructions. In brief, after the indicated treatment, NP cells were fixed with 0.2% glutaraldehyde for 15 min at 37 °C. After washing with PBS, the cells were stained with freshly prepared X-gal staining solution (pH 6.0) at 37 °C overnight. After overnight incubation, the cells were washed with PBS and visualized under a microscope.

### ER-Tracker and LysoTracker staining

ER-Tracker Green and LysoTracker Red staining were utilized to assess the extent of ER and lysosome colocalization. In brief, cells were washed with Hank’s balanced salt solution (HBSS) and then stained with 1 µM ER-Tracker Green and 50 nM LysoTracker Red at 37 °C for 30 min. Nuclei were stained with Hoechst 33342 (Beyotime) for 10 min. After staining, the cells were washed with PBS and observed with a fluorescence microscope (Olympus, BX53; Melville, NY, USA).

### Animal model

A total of 18 male Sprague‒Dawley (SD) rats (3-months old, weighing 300–400 g) were obtained from the Laboratory Animal Center of Tongji Medical College, Huazhong University of Science and Technology (Wuhan, China) and housed under strictly controlled environmental conditions. The rat models of IDD were established as previously described^31,32^. The SD rats were randomly assigned to three groups: a control group, an IDD group, and an IDD + TMG group. An operator blinded to the grouping performed the experiment. The rats were anesthetized with sodium pentobarbital (40 mg/kg), and the experimental disc level (Co7/8) in the coccygeal vertebrae of each rat was located by digital palpation and confirmed by trial radiography. After skin sterilization, a syringe needle (27-G) was used to puncture the annulus fibrosus layer (~4 mm in depth from the skin) along the vertical direction. The syringe needle was rotated in the axial direction by 360° and held in place for 30 s. Subsequently, 2 μL of PBS and 2 μL of TMG (10 μM) were slowly injected into the NP cavity of the rats in the IDD group and IDD + thiamet G (TMG) group, respectively. All rats were allowed to engage freely in activity and were monitored every day to ensure their well-being. Thereafter, the injections were repeatedly performed every two weeks with a smaller needle (31-G) to minimize annulus fibrosus injury. Eight weeks after surgery, all rats were anesthetized and subjected to MRI examination using an MR scanner (Bruker BioSpec70/20USR, Bruker, Germany), and serial sagittal T2-weighted MR images were assessed to grade the degree of IDD on the basis of the Pfirrmann classification^30^. Then, all rats were euthanized, and the corresponding intervertebral disc tissues were harvested for further analysis.

### Histological and histochemical analysis

The harvested intervertebral disc specimens were fixed with 4% paraformaldehyde at room temperature for 48 h and decalcified with 10% ethylenediaminetetraacetic acid (EDTA; pH 7.4) at 37 °C for 30 days. Then, the specimens were embedded in paraffin blocks and sectioned at a thickness of 5 μm along the midsagittal orientation. For hematoxylin and eosin (HE) and safranin O-fast green (S-O) staining, all procedures were performed according to the manufacturer’s guidance (Solarbio, Beijing, China). Histological scores were determined as previously described (<5, normal intervertebral discs; 6–11, moderate IDD; and 12–14, severe IDD)^33^.

For immunohistochemical experiments, the sliced sections were incubated with primary antibodies against O-GlcNAc (diluted 1:100), FAM134B (diluted 1:150) and p16 (diluted 1:150) at 4 °C overnight. After washing three times with PBS, the sections were incubated with the appropriate secondary antibodies at room temperature for 50 min and counterstained with hematoxylin. Then, the sections were imaged and analyzed with a digital slide scanner (S360, Hamamatsu, Japan). Furthermore, apoptosis was assessed using a TUNEL apoptosis assay kit (Yeasen) according to the manufacturer’s instructions, and images were acquired with a fluorescence microscope (Olympus IX71).

### Statistical analysis

Statistical analyses were performed with Prism statistical software (version 8.0; GraphPad). All data are presented as the mean ± SD of at least three independent experiments. The distribution of data was assessed for normality by Kolmogorov–Smirnov test. Student’s *t* test and one-way analysis of variance (ANOVA) followed by Tukey’s test were performed for comparisons between two experimental groups and more than two experimental groups, respectively, where applicable. Nonparametric data (human samples, Pfirrmann grades and histological scores) were analyzed by Mann‒Whitney *U* test. A *P* value < 0.05 was considered to be statistically significant.

More materials and methods are available in the Supporting Information.

## Results

### Enhanced OGT expression and O-GlcNAcylation in human degenerated NP tissues and nutrient-deprived human NP cells

**Fig. 1:**
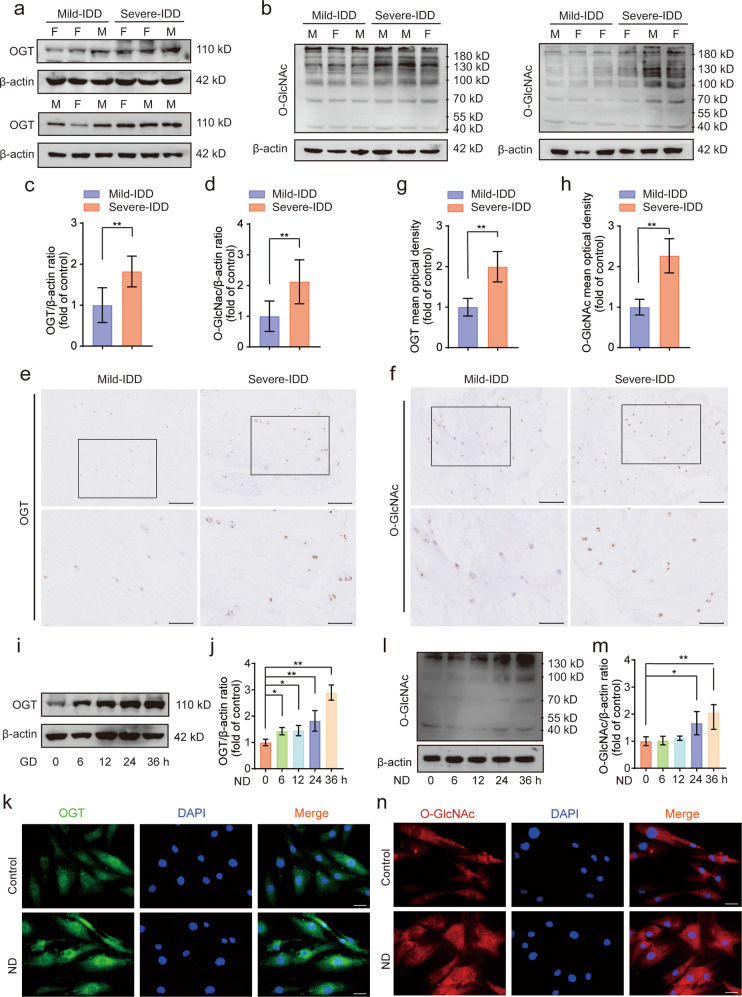
Enhanced expression of OGT and O-GlcNAc in degenerated human NP tissues and nutrient-deprived NP cells. **a**–**d** Western blot analysis showing the protein expression levels of OGT and O-GlcNAc in human NP cells isolated from NP tissues. The relative band densities were quantified. The two groups were matched by sex. M: male, F: female; *n* = 12. **e**–**h** Representative images showing IHC staining for OGT and O-GlcNAc in human NP tissues. Typical staining in the boxed areas of the images is magnified and shown in the lower rows. The relative mean optical densities were quantified; *n* = 12. **(i**, **j**, **l**, **m)** NP cells were subjected to ND for different durations (0, 6, 12, 24, and 36 h). Western blots showing the levels of OGT protein expression and O-GlcNAc. The relative band densities were quantified. **k**, **n** After treatment with ND for 36 h, NP cells were labeled with anti-OGT and anti-O-GlcNAc antibodies, and representative fluorescence images are shown. Nuclei were stained with DAPI; scale bar: 50 μm. The data are presented as the mean ± SD values. ^∗∗^*P* < 0.01, ^∗^*P* < 0.05.

To investigate the role played by O-GlcNAcylation and its clinical relevance in IDD, we collected six mildly degenerated and six severely degenerated NP tissues, which were selected based on preoperative MRI scans on the basis of the Pfirrmann classification system. The relative protein expression levels of OGT and O-GlcNAc in these tissues were determined by performing western blot analysis and immunohistochemical staining. As demonstrated in Fig. 1a–d, the global levels of OGT in the severely degenerated human NP tissues were significantly higher than those in the mildly degenerated NP tissues, and the expression of OGA in the severely degenerated NP tissues was higher than that in the mildly degenerated NP tissues (Supplementary Fig. 1a, b). Similarly, significantly higher O-GlcNAc modification abundance was observed in severely degenerated human NP tissues than in mildly degenerated NP tissues (Fig. 1e–h). No sex difference was identified in the expression of OGT or O-GlcNAc between the two groups. Furthermore, nutrient deficiency has been positively associated with the pathogenesis of IDD, and OGT has been established as a nutrient sensor that functions primarily via its posttranslational modulation in response to cellular nutrient fluctuations. Thus, in our study, human NP cells were subjected to ND treatment for different durations (0, 6, 12, 24, and 36 h) to establish a cell injury model (Supplementary Fig. 2a). As shown in the western blot results presented in Fig. 1i, j, l, and m, compared to those in the control group, the levels of OGT protein expression and O-GlcNAc were markedly increased in a time-dependent manner in human NP cells after ND treatment. In addition, as shown in the immunofluorescence results presented in Fig. 1k, n, compared with the effects on the control group, ND treatment significantly elevated the fluorescence intensities of OGT and O-GlcNAc. Collectively, these results suggested that OGT activity and O-GlcNAc modification were likely correlated with the occurrence of IDD.

### OGT regulated human NP cell senescence and apoptosis under ND conditions

**Fig. 2:**
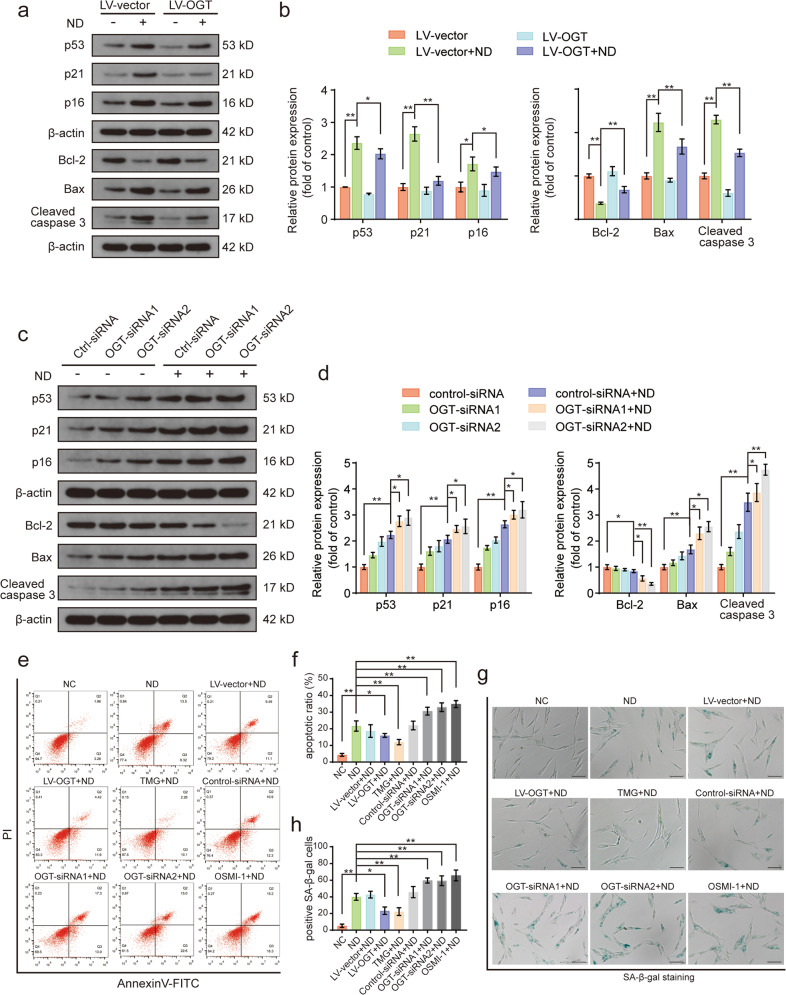
OGT modulates apoptosis and senescence in nutrient-deprived human NP cells. **a**, **b** NP cells were transduced with the OGT overexpression lentiviral vector or empty vector for 72 h and were then subjected to ND for 36 h. The senescence- and apoptosis-associated proteins p53, p21, p16, Bcl-2, Bax, and cleaved caspase 3 were evaluated via western blot analysis, and the relative band densities were quantified. **c**, **d** After transfection with siRNA for 48 h, NP cells were subjected to ND for 36 h. Western blots showing the protein expression levels of senescence- and apoptosis-associated proteins p53, p21, p16, Bcl-2, Bax, and cleaved caspase3, and the relative band densities were quantified. **e**, **f** Representative flow cytometry dot plot of cells subjected to Annexin V/PI double staining for assessment of apoptosis. Annexin V+/PI− cells and Annexin V+/PI+ cells were quantified as the total apoptotic cell population. TMG, a specific OGA inhibitor. OSMI-1, a specific OGT inhibitor. **g**, **h** SA-β-gal staining results showing NP cell senescence. Representative images of SA-β-gal staining with the proportions of positive cells illustrated; scale bar: 100 μm. The data are presented as the mean ± SD values. ^∗∗^*P* < 0.01, ^∗^*P* < 0.05.

Recent evidence has confirmed the fundamental role played by O- the GlcNAcylation in regulating cell functional activity and survival in response to various stress conditions^23,34,35^. We therefore evaluated the effects of the O-GlcNAc modification on apoptosis and senescence by genetically and pharmacologically modulating the expression of OGT in human NP cells. First, OGT was successfully overexpressed and knocked down in human NP cells via lentiviral transduction and siRNA transfection, respectively (Supplementary Fig. 3a–d). Moreover, the expression of the apoptosis- and senescence-associated proteins p53, p21, p16, Bcl-2, Bax, and cleaved caspase 3 were analyzed by western blotting, and the data showed that nutrient-deprived human NP cells exhibited increased expression of apoptotic and senescence-associated proteins relative to the control group, whereas OGT overexpression significantly attenuated this increase (Fig. 2a, b). However, OGT knockdown by OGT siRNA transfection exacerbated the ND-stimulated upregulated expression of apoptosis- and senescence-associated proteins, suggesting that OGT downregulation promoted the apoptosis- and senescence-inducing effects of ND on human NP cells (Fig. 2c, d). In addition, we found that upregulation of O-GlcNAcylation induced by OGA knockdown clearly attenuated ND-induced apoptosis and senescence in NP cells (Supplementary Fig. 4a–f). Furthermore, we employed annexin V-PI double staining and SA-β-gal staining to confirm the protective antiapoptotic and antisenescence effects of O-GlcNAcylation in cells under ND conditions. TMG and OSMI-1 are two well-characterized pharmacological inhibitors of OGA and OGT, respectively, that can modulate the global level of cellular O-GlcNAc modification. Both TMG and OSMI-1 showed no obvious cytotoxic effects on human NP cells (Supplementary Fig. 2b, c). As shown by the flow cytometry results presented in Fig. 2e, f, a significant increase in the apoptotic cell-to-live cell ratio was observed in the ND group compared with that in the normal control group. OGT overexpression and TMG treatment efficiently alleviated ND-induced apoptosis, but OGT knockdown and OSMI-1 treatment significantly increased the ND-induced apoptosis rate. Consistent with these findings, the SA-β-gal staining results demonstrated that the quantitative increase in the SA-β-gal-positive cell population observed in the ND group was profoundly decreased after OGT overexpression or TMG treatment, while OGT knockdown or OSMI-1 treatment abrogated the protective effects of O-GlcNAcylation against ND-induced cellular senescence (Fig. 2g, h). Therefore, our results demonstrated that OGT regulated the apoptosis and senescence rates in human NP cells under ND conditions.

### OGT modulated FAM134B-mediated ER-phagy upon ND

**Fig. 3:**
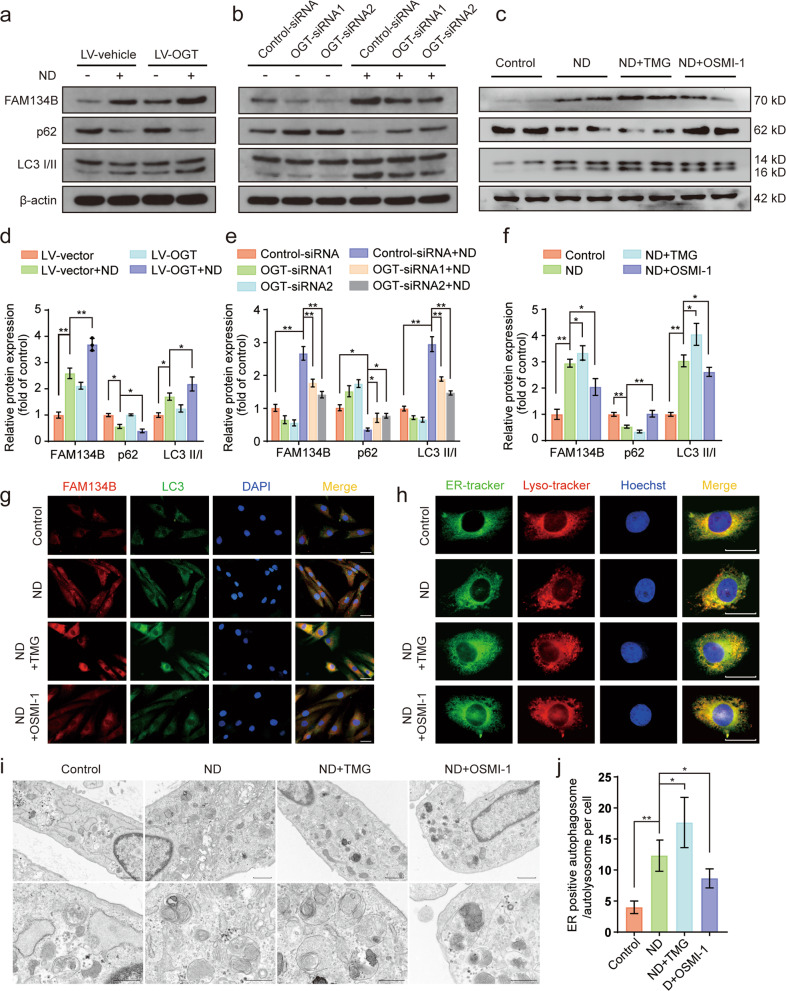
Upregulation of O-GlcNAcylation facilitates FAM134B-mediated ER-phagy in nutrient-deprived NP cells. **a**–**f** NP cells were transfected with OGT-overexpressing lentivirus for 72 h or OGT siRNA for 48 h and were then subjected to ND for 36 h or directly subjected to ND in the presence of TMG and OSMI-1 for 36 h. Western blot showing the protein expression levels of FAM134B, LC3, and p62, and the relative band densities were quantified. **g** NP cells were subjected to ND combined with TMG or OSMI-1 treatment for 36 h. The relative expression and subcellular distribution of FAM134B and LC3 were determined by double immunofluorescence staining; scale bar: 50 μm. **h** NP cells were treated as indicated in **g**. The ER and lysosomes were labeled with the specific dyes ER-Tracker Green and LysoTracker Red, respectively. Representative images are shown; scale bar: 20 μm. **i**, **j** NP cells were treated as indicated in **g**. Autophagosomes/autolysosomes containing ER fragments or whorls were evaluated by transmission electron microscopy; scale bars: 1 μm and 500 nm. The data are presented as the mean ± SD values. ^∗∗^*P* < 0.01, ^∗^*P* < 0.05.

FAM134B-mediated ER-phagy is essential for maintaining ER homeostasis and quality control because it degrades excessive ER fragments and misfolded ER-resident proteins under different stress conditions, such as starvation. O-GlcNAcylation has been implicated in differences in the spatiotemporal regulation of autophagy^26,28^. However, the relationship between O-GlcNAcylation and FAM134B-mediated ER-phagy, specifically in human NP cells, has been largely unclear. We therefore investigated the influence of O-GlcNAcylation on FAM134B-mediated ER-phagy in human NP cells under ND conditions. First, the levels of ER phagy-related proteins FAM134B, LC3 and p62 were measured by western blotting. As shown in the data presented in Fig. 3a–f, compared with that in the normal control group, significantly increased protein expression of FAM134B and LC3 was observed under ND conditions and was accompanied by downregulation of p62 protein expression, suggesting that FAM134B-mediated ER-phagy was elicited by ND. In addition, OGT overexpression and TMG treatment further enhanced the protein expression of FAM134B and LC3 and reduced p62 protein expression. In contrast, OGT knockdown and OSMI-1 treatment partially counteracted the ND-induced upregulation of FAM134B and LC3 expression, and this attenuated effect was accompanied by a moderate increase in p62 expression. Similarly, when compared to that of the ND treatment alone, the effect of upregulated O-GlcNAcylation after OGA knockdown triggered notably increased FAM134B and LC3 protein expression levels and decreased p62 expression levels (Supplementary Fig. 5a, b). Moreover, immunofluorescence assays confirmed that ND treatment increased FAM134B and LC3 fluorescence intensities compared with those in the control group; in addition, the fluorescence intensities and colocalization of FAM134B and LC3 were significantly increased after TMG treatment and significantly decreased after OSMI-1 treatment (Fig. 3g). Moreover, to visually monitor ER-phagy, ER-Tracker Green and LysoTracker red were used to label the ER and lysosomes, respectively. As shown in Fig. 3h, ND induced an obvious punctate ER staining pattern relative to the pattern in the control group, and these puncta were mostly colocalized to lysosome-stained cellular areas. Moreover, TMG treatment promoted and OSMI-1 treatment attenuated ND-induced ER fragmentation and punctate colocalization with lysosomes. In addition, we next detected the formation of autophagosomes/autolysosomes containing ER structures via transmission electron microscopy to verify the occurrence of ER-phagy. As shown in the results presented in Fig. 3i, j, the increase in the number of autophagosomes/autolysosomes containing ER fragments under ND conditions was markedly enhanced after TMG treatment and was partially attenuated after OSMI-1 treatment. Taken together, these data demonstrated that FAM134B-mediated ER-phagy was positively correlated with O-GlcNAc modification.

### FAM134B-mediated ER-phagy was involved in the protective effects of OGT in response to ND

**Fig. 4:**
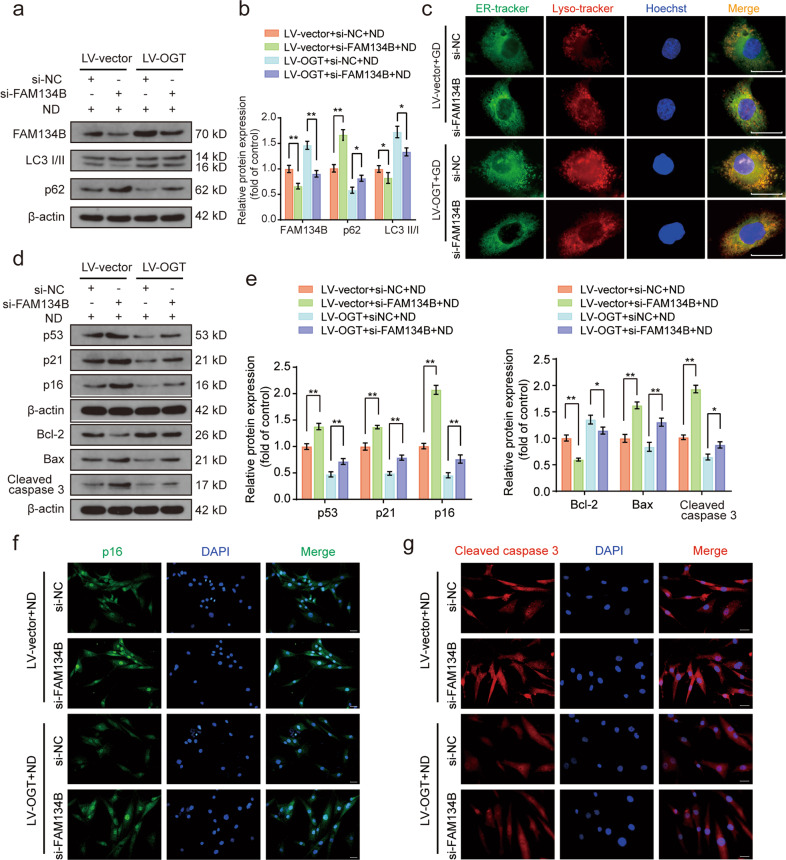
FAM134B-mediated ER-phagy inhibition partially attenuates the protective effect of OGT against ND. **a**, **b** After transfection with FAM134B-siRNA for 48 h, OGT-overexpressing NP cells were subjected to ND for 36 h. Western blot analysis showing the protein expression levels of FAM134B, LC3, and p62, and the relative band densities were quantified. **c** NP cells were handled as indicated in **a**. Representative results of ER-Tracker Green and LysoTracker Red staining showing the colocalization profiles of the ER and lysosomes; scale bar: 20 μm. **d**, **e** NP cells were handled as indicated in **a**. Western blot analysis showing the protein expression levels of p53, p21, p16, Bcl-2, Bax, and cleaved caspase 3, and the relative band densities were quantified. **f**, **g** NP cells were treated as indicated in **a**. Representative immunofluorescence results showing p16 and cleaved caspase 3 expression in NP cells. Nuclei were stained with DAPI; scale bar: 100 μm. The data are presented as the mean ± SD values. ^∗∗^*P* < 0.01, ^∗^*P* < 0.05.

Next, we investigated whether FAM134B-mediated ER-phagy is a key event in the protective effects of OGT in response to ND. FAM134B siRNA was used to downregulate FAM134B-mediated ER-phagy activation. We transfected FAM134B siRNA into human NP cells and verified its knockdown efficiency by western blotting (Supplementary Fig. 6a, b). As shown in the western blot data presented in Fig. 4a, b, OGT overexpression markedly upregulated the protein expression levels of FAM134B and LC3, and this increase was accompanied by a decrease in the p62 level. In addition, FAM134B knockdown significantly weakened the effects of OGT overexpression on ER-phagy, suggesting that FAM134B knockdown profoundly attenuated ER-phagy activity in response to ND and OGT overexpression. Similarly, ER and lysosome staining indicated that FAM134B knockdown significantly suppressed ER clustering and colocalization with lysosomes resulting from OGT overexpression (Fig. 4c). In addition, western blot analysis revealed that OGT overexpression significantly reduced the levels of apoptosis-associated proteins (Bcl-2, Bax, and cleaved caspase 3) and senescence-associated proteins (p53, p21, and p16) in response to ND, while the protective effects of O-GlcNAcylation under ND conditions were obviously diminished by FAM134B knockdown (Fig. 4d, e). Immunofluorescence staining of p16 and cleaved caspase 3 showed that the fluorescence intensities of p16 and cleaved caspase 3 under ND conditions were weakened by OGT overexpression, while FAM134B knockdown discernibly reversed the effects of OGT overexpression (Fig. 4f, g). These results indicated that FAM134B-mediated ER-phagy activation was closely associated with the protective effects of OGT in ND-induced human NP cell injury.

### OGT interacted with and stabilized FAM134B

**Fig. 5:**
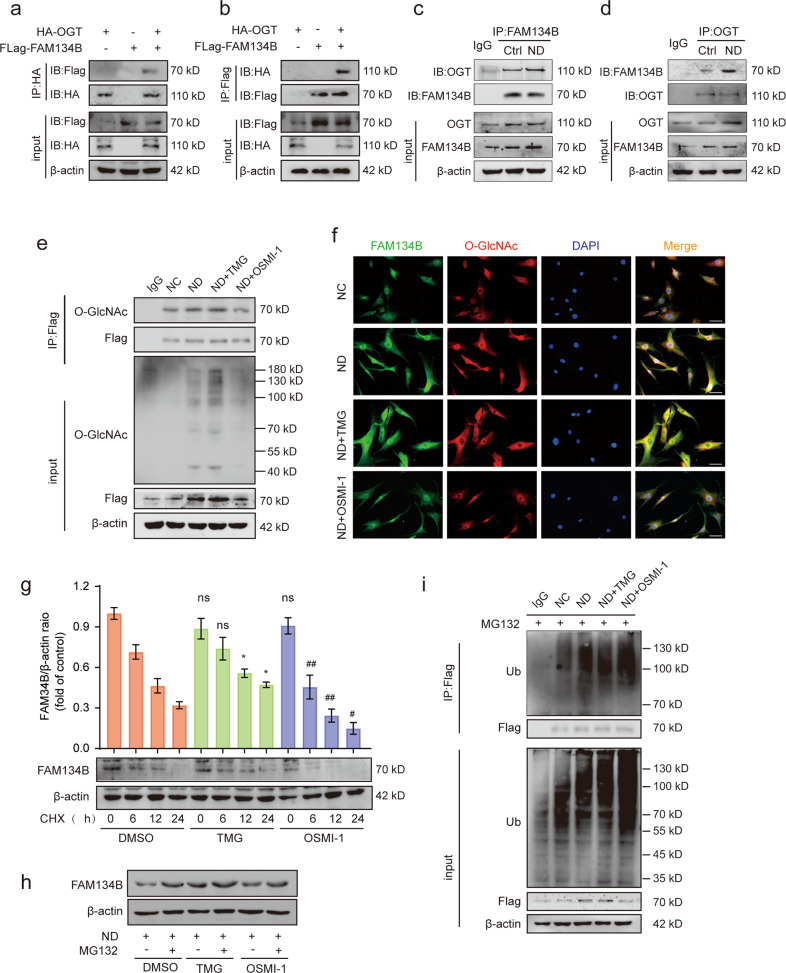
OGT interacts with and stabilizes FAM134B. **a**, **b** NP cells were cotransfected with HA-OGT and Flag-FAM134B. Whole-cell lysates were subjected to immunoprecipitation with anti-Flag or anti-HA antibodies, and the precipitates were then immunoblotted with anti-Flag and anti-HA antibodies to show the interaction between HA-OGT and Flag-FAM134B. **c**, **d** NP cells were subjected to the indicated treatment, and the endogenous interaction between OGT and FAM134B was detected via coimmunoprecipitation. Then, the anti-OGT and anti-FAM134B immunoprecipitates were immunoblotted with anti-OGT and anti-FAM134B antibodies, respectively. **e** Flag-FAM134B-transfected NP cells were subjected to ND in the presence of TMG or OSMI-1 for 36 h. Cell lysates were immunoprecipitated with an anti-Flag antibody and then immunoblotted with anti-Flag and anti-O-GlcNAc antibodies. **f** NP cells were subjected to treatment as indicated in **e**. Colocalization of endogenous FAM134B and O-GlcNAc was visualized by immunofluorescence staining. **g** After pretreatment with TMG or OSMI-1 for 24 h, NP cells were treated with CHX (50 µg/mL) for the indicated duration. The cells were harvested, and the FAM134B protein level was measured by western blot analysis. **h** NP cells were subjected to ND treatment in the presence of TMG and OSMI-1 for 36 h, and the proteasome inhibitor MG132 (10 µM) was added 6 h before cell collection. The protein level of FAM134B was assessed via western blot analysis. **i** Flag-FAM134B-transfected NP cells were subjected to ND in the presence of TMG or OSMI-1 for 36 h, and the proteasome inhibitor MG132 (10 µM) was added 6 h before cell collection. Anti-Flag immunoprecipitates of denatured proteins from cell lysates were then analyzed by western blotting with an anti-ubiquitin antibody. The data are presented as the mean ± SD values. ^∗∗^*P* < 0.01, ^∗^*P* < 0.05.

OGT is the only known cytonuclear O-GlcNAc deposition enzyme, and it regulates various target proteins typically by catalyzing their O-GlcNAcylation. We thus examined whether FAM134B is O-GlcNAcylated by OGT. HA-tagged OGT and Flag-tagged FAM134B were cotransfected into human NP cells, and coimmunoprecipitation (co-IP) experiments were performed. Ectopically expressed HA-tagged OGT was detected in the Flag-tagged FAM134B precipitate, and Flag-tagged FAM134B was found in the HA-tagged OGT precipitate (Fig. 5a, b). More importantly, an interaction between endogenous OGT and FAM134B in human NP cells was identified via reciprocal immunoprecipitation using anti-OGT and anti-FAM134B antibodies; endogenous OGT directly interacted with FAM134B, and notably, ND treatment appreciably enhanced this interaction (Fig. 5c, d). Furthermore, to confirm the physiological interaction, we transfected human NP cells with Flag-tagged FAM134B and performed coimmunoprecipitation with an anti-Flag antibody. The results demonstrated that ND notably increased the O-GlcNAcylation of FAM134B. This interaction was further enhanced with TMG treatment and considerably weakened with OSMI-1 treatment (Fig. 5e). Immunofluorescence staining for FAM134B and O-GlcNAc corroborated their highly consistent spatial colocalization (Fig. 5f). Given that OGT interacts with and stabilizes FAM134B, we assessed the half-life of FAM134B by blocking nascent protein synthesis with CHX. TMG pretreatment greatly delayed FAM134B protein degradation compared with that in the control group, while OSMI-1 pretreatment exerted the opposite effect (Fig. 5g). In addition, marked protein accumulation of FAM134B was observed in all groups in the presence of the proteasome inhibitor MG132, suggesting that OGT may stabilize FAM134B by influencing its ubiquitination (Fig. 5h). Then, immunoprecipitation was performed with an anti-ubiquitin antibody to assess the extent of FAM134B ubiquitination. ND significantly increased the ubiquitination level of FAM134B, while upregulation of O-GlcNAcylation by TMG treatment partially attenuated the ubiquitination of FAM134B (Fig. 5i). These results indicated that OGT can O-GlcNAcylate FAM134B and promote its protein stability by inhibiting its ubiquitination-mediated degradation.

### Administration of TMG partially ameliorated IDD in vivo

**Fig. 6:**
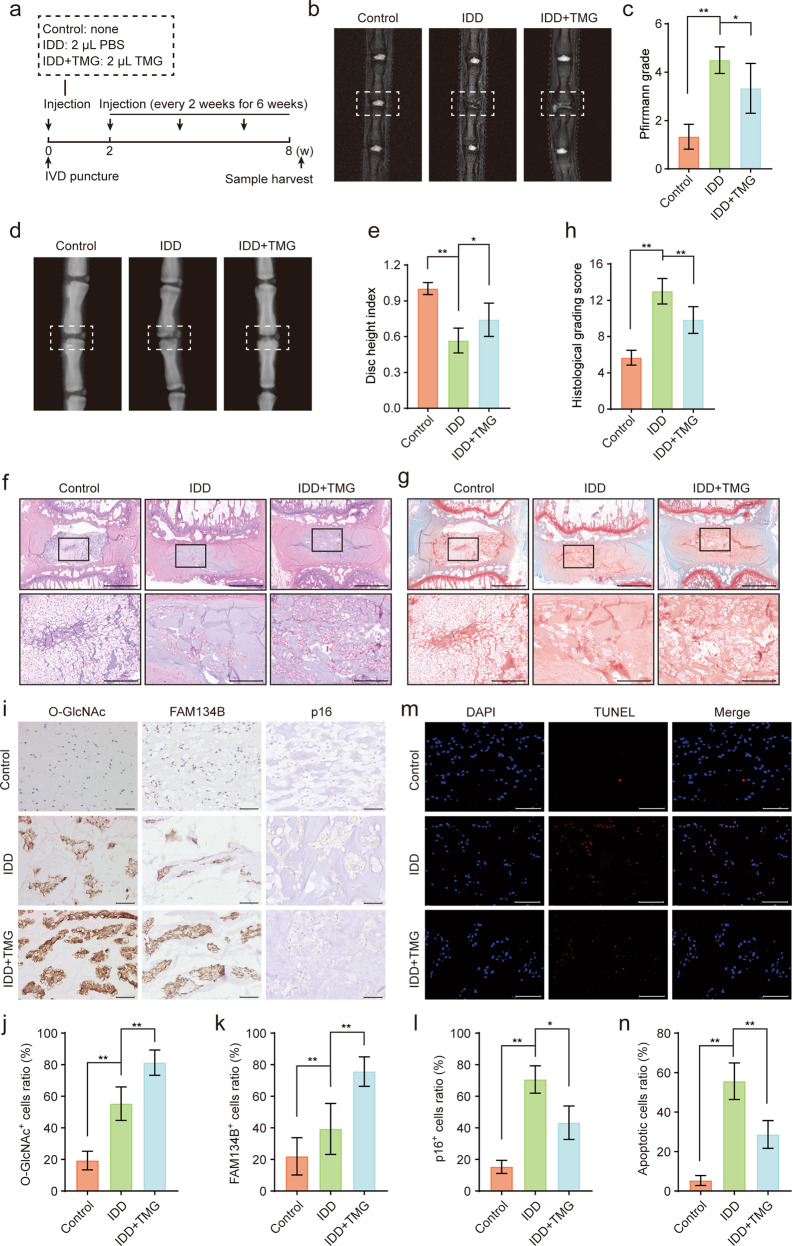
Administration of TMG partially ameliorates IDD in rats. **a** Schematic illustration of the animal model construction and treatments in this study. **b**, **c** After 8 weeks of the indicated treatment, the degree of disc degeneration of rat caudal discs was evaluated by T2-weighted MRI and the corresponding Pfirrmann MRI grades were determined. Representative images are shown. **d**, **e** Changes in rat caudal disc height were assessed by X-ray imaging. Representative X-ray images were acquired and evaluated on the basis of the DHI. **f**–**h** Representative images showing HE and S-O staining of midsagittal sections of rat disc specimens and the corresponding quantitative histologic scores; scale bars: 1 mm and 250 μm. **i**–**l** Representative images of O-GlcNAc, FAM134B and p16 immunohistochemical staining of midsagittal sections of rat disc specimens. The immunopositively stained cell to total cell ratio was calculated. Scale bar: 100 μm. **m**, **n** Representative images of TUNEL in midsagittal sections of rat disc specimens. Nuclei were stained with DAPI, and positive TUNEL-stained cells were quantified; scale bar: 200 μm. The data are presented as the mean ± SD values. ^∗∗^*P* < 0.01, ^∗^*P* < 0.05.

In vitro experiments showed that the upregulation of O-GlcNAcylation suppressed NP cell apoptosis and senescence by promoting FAM134B-mediated ER-phagy. We further explored the therapeutic effects of upregulating O-GlcNAcylation in vivo. TMG has been demonstrated to be a specific, stable, permeable, potent, and efficient inducer of O-GlcNAcylation, as indicated by its inhibition of OGA catalytic activity in multiple cell types and tissues. In this study, eight weeks after surgery, all rats were euthanized (Fig. 6a), and the extent of disc degeneration was evaluated via MRI and X-ray examination. As shown in the results presented in Fig. 6b−e, a heterogeneous and decreased MRI T2-weighted signal was found for the IDD group, and disc height on X-rays was reduced relative to that in the control group. These alterations were partially reversed by TMG administration, and the quantitative Pfirrmann grade and disc height index (DHI) corroborated these qualitative observations. In addition, morphological and histological changes in intervertebral discs were detected by HE and S-O staining, which showed that the IDD group exhibited markedly disorganized collagen arrangement within lamella and inward bulging of the annulus fibrosus, an unclear boundary between the NP and the annulus fibrosus, reduced NP volume, and NP cell loss. In contrast, additional TMG administration partially alleviated the degenerative changes in intervertebral discs (Fig. 6f, g), and the quantitative histological score indicated that TMG retarded IDD development (Fig. 6h). Moreover, the immunohistochemical staining results showed that the O-GlcNAc and FAM134B levels were increased in the IDD group compared with the control group and were further increased by TMG treatment (Fig. 6i–k). Finally, immunohistochemical staining of p16 and TUNEL assays were performed to characterize the protective effects of TMG on senescence and apoptosis in rat NP tissue, and the results suggested that the proportions of p16- and TUNEL-positive cells were markedly increased in the IDD group and notably decreased in the TMG cotreatment group (Fig. 6i, l–n). Collectively, these results demonstrated that upregulation of O-GlcNAcylation induced by TMG treatment may have partially retarded IDD progression by modulating FAM134B-mediated ER-phagy.

## Discussion

**Fig. 7:**
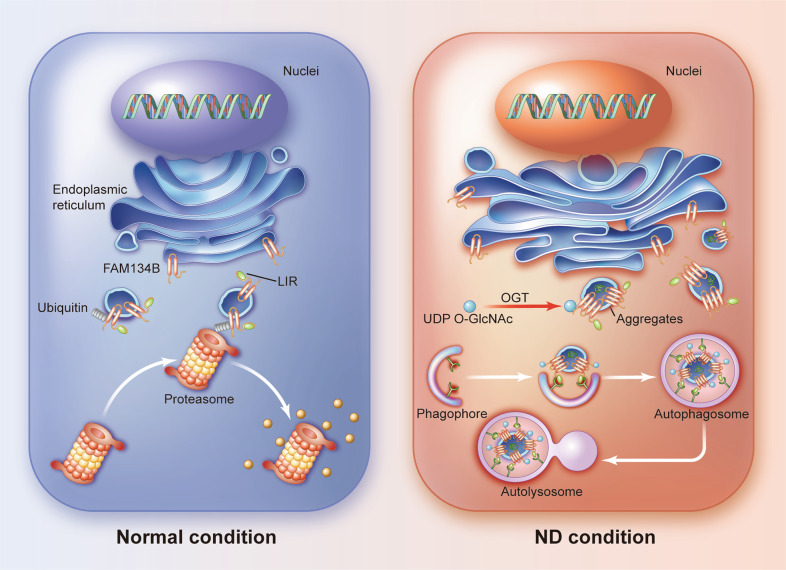
Schematic illustration showing the possible mechanisms by which OGT modulates FAM134B-mediated ER-phagy and promotes cell survival. Under ND conditions, FAM134B is possibly O-GlcNAcylated by OGT, which suppresses its ubiquitination-mediated degradation. Subsequently, enhanced FAM134B-mediated ER-phagy restores intracellular homeostasis and facilitates cell survival.

Emerging evidence indicates that OGT is generally associated with a variety of pathophysiological processes because it alters the degree of O-GlcNAcylation of target proteins, and aberrant O-GlcNAcylation homeostasis directly leads to the occurrence and progression of multiple human disorders^23,25^. Therefore, further investigation into the role played by O-GlcNAcylation in IDD is a meaningful research direction. In the present study, elevated OGT expression and O-GlcNAcylation were found in degenerated NP tissues and nutrient-deprived NP cells. Enhanced O-GlcNAcylation induced by genetic manipulation or pharmacological treatment was clearly associated with decreased NP cell apoptosis and senescence in response to ND. Mechanistically, FAM134B, the typical ER-phagy receptor, was identified as a target of OGT, and O-GlcNAcylation of FAM134B markedly reduced ubiquitination-mediated degradation of FAM134B, enhancing cell survival under ND conditions (Fig. 7). Furthermore, the results of our in vivo experiments suggested that abnormal O-GlcNAcylation levels were closely correlated with the onset of IDD.

O-GlcNAc modification has been clearly established as an important regulatory mechanism involved in gene transcription, protein synthesis, cell metabolism, Ca^2+^ handling, and cell survival. To date, OGT and OGA are the only enzymes known to catalyze O-GlcNAc deposition and removal, respectively, of target proteins; specifically, OGT and OGA rapidly and dynamically orchestrate intracellular O-GlcNAcylation homeostasis in response to diverse intrinsic and extrinsic stimuli, including changes in nutrient availability and the metabolic state, increased oxidative stress, and aging^24^. Specifically, OGT is expressed mainly as three isoforms: nucleocytoplasmic OGT (ncOGT), mitochondrial OGT (mOGT) and short OGT (sOGT), which differ in length, subcellular distribution, and biological roles^23^. ncOGT is the canonical type and is present mainly in the nucleus and cytoplasm, where it is linked to gene transcription, proteasomal degradation, and stress tolerance. Moreover, an increasing number of studies have indicated that mOGT and sOGT are expressed in certain human cell lines and tissues and exert specific biofunctions, such as energy production and cell survival^24^. Intensive research focused on O-GlcNAcylation functions is currently being conducted to gain insight into disease etiology and therapeutic targets. In the brain, optimal O-GlcNAcylation homeostasis has been associated with the maintenance of intrinsic neuronal properties, including neuronal growth, maturation, and synaptic plasticity, under physiological conditions^36,37^. In addition, relatively low O-GlcNAcylation levels have been found in patients with Alzheimer’s disease (AD), and genetic or pharmacological downregulation of O-GlcNAcylation has led to increased neuron loss, Aβ aggregation, tau hyperphosphorylation, and even learning and memory deficits in mice^35,38,39^. In models of cardiac infarction, ischemia‒reperfusion, and diabetic cardiac disorders, an acute increase in the O-GlcNAcylation level contributed to improved cardiac survival by facilitating adaptive mechanistic processes, such as mitochondrial function, the ER stress response, and autophagy, although chronic accumulation of O-GlcNAc marks within the heart has been suggested to be correlated with cardiac dysregulation^25,40,41^. After viral infection, increased glucose metabolism and O-GlcNAc levels were found to be essential for O-GlcNAcylation and K63-linked ubiquitination of MAVS and for the initiation of downstream antiviral innate immune signaling in immune cells^42^. Because dysregulation of O-GlcNAcylation is highly involved in the onset and progression of multiple diseases, we the investigated O-GlcNAcylation rate in intervertebral discs and found elevated O-GlcNAcylation levels in degenerated human NP tissues. Moreover, increasing the O-GlcNAcylation rate through specific genetic manipulation or pharmacological intervention greatly increased NP cell survival and function under nutrient deprivation conditions. Therefore, based on these findings, a therapeutic approach targeting O-GlcNAcylation may provide a new solution for the treatment of IDD.

ER-phagy, a recently characterized type of selective autophagy, plays a core role in ER protein quality control and ER homeostasis maintenance through selective degradation of excessive ER membrane fragments and ER lumen-resident protein aggregates, especially aggregates of specific proteins, such as procollagens, that are not cleared by ERAD^43^. Normally, ER-phagy activation is a critical adaptive mechanism for maintaining ER homeostasis under multiple detrimental conditions, such as nutrient starvation and calcium homeostasis disruption, while defects in ER-phagy may result in the pathogenesis of numerous disorders. FAM134B was the first ER membrane protein found to be involved in delivering ER fragments for lysosomal degradation. FAM134B mutation-induced ER-phagy suppression markedly compromised sensory and autonomic neurons survival and activity and thus promoted the progression of neurological disorders^44,45^. FAM134B-mediated ER-phagy activation was implicated in the restriction of Ebola, dengue and Zika virus replication, and cells with FAM134B depletion showed a substantial increase in viral RNA^21,46^. Interestingly, FAM134B-mediated excessive ER degradation has been reported to impede ER homeostasis and result in increased ER stress and subsequent apoptosis of HeLa cells^47^. In addition, in an AT-1 transgenic mouse model of progeria, increased transport of cytosolic acetyl‐CoA into the ER lumen and blockade of ER autophagic recycling were found to be closely linked to defective ER-phagy, and functional restoration of ER-phagy rescued the aging phenotype in total and extended the lifespan^48^. Furthermore, it has been established that autophagy is crucial for the maintenance of IVD homeostasis and that autophagy impairment is directly involved in the induction of irregular notochordal cell disappearance, nonnotochordal cell apoptosis and senescence^49–51^. Therefore, we investigated the effects of FAM134B-mediated ER-phagy on cell functionality and survival in human NP cells under nutrient starvation conditions and found that FAM134B-mediated ER-phagy was activated in response to ND and that FAM134B knockdown significantly aggravated ND-induced apoptosis and senescence. These findings indicated that FAM134B-mediated ER-phagy was enhanced in nutrient-deprived NP cells and exerted a cytoprotective effect.

Both O-GlcNAcylation and autophagy are extremely conserved adaptive regulatory mechanisms that are sensitive to stress and nutrient availability. Increasing evidence suggests a strong intrinsic association between O-GlcNAcylation and autophagy. Unc-51-like kinase 1 (ULK1), a key enzyme required for phagophore formation, has been revealed to be O-GlcNAcylated by OGT during the initiation stage of autophagy and to subsequently interact with ATG14L and VPS34 to trigger autophagy initiation under glucose starvation conditions^52^. In starving HEK293T cells and mouse livers, O-GlcNAc was crucial for autophagy activation and glucose homeostasis^53^. In contrast, a few studies reported that hyper-O-GlcNAcylation hindered autophagic activity and the fusion of autophagosomes with lysosomes in *Caenorhabditis elegans* and Drosophila^27,54^. In addition, in cortical astrocytes and cardiomyocytes, both reduced and increased O-GlcNAcylation levels were positively correlated with autophagic activity^55,56^. These findings may suggest that autophagy is finely tuned by O-GlcNAcylation in a context- and cell-dependent manner. Studying human NP cells, we found that increased O-GlcNAcylation by OGT enhanced FAM134B-mediated ER-phagy by promoting FAM134B protein stability. Taken together, these results indicated that independent of factors such as species, cell type, tissue heterogeneity, experimental design, basic metabolic condition, or target protein, O-GlcNAcylation signaling exerted divergent effects on autophagic flux, suggesting that increasing and decreasing O-GlcNAcylation may not lead to opposite outcomes. However, maintaining O-GlcNAcylation at an optimal level is crucial for cellular function and survival, and targeting O-GlcNAcylation homeostasis is a promising approach for disease prevention and treatment.

Although our findings strongly suggest a close relationship between O-GlcNAcylation and FAM134B-mediated ER-phagy, site mapping is the most decisive method for confirming protein O-GlcNAcylation. This aspect of our study needs to be substantiated by further research. In addition, NP cells isolated from human NP tissues represent a heterogeneous population consisting mainly NP cells with a certain percentage of other cell subsets, such as notochordal cells. In addition, the puncture injury-induced IDD model in rats only partially mimics the natural degenerative process in the human intervertebral disc and cannot exactly match the damage pattern evident in our in vitro experiment. Therefore, verification of the therapeutic efficacy may require a more appropriate animal model and a longer surveillance period. Furthermore, the therapeutic outcomes of pharmacological administration alone are still preliminary and may be more reliable and convincing when combined with appropriate genetic approaches.

In summary, our findings demonstrated that transient upregulation of O-GlcNAcylation was beneficial for the function and survival of human NP cells under ND conditions and retarded the progression of IDD in rats. FAM134B was identified as a possible target of OGT, and FAM134B O-GlcNAcylation was found to enhance FAM134B protein stability and promote subsequent ER-phagy. Therefore, the regulation of O-GlcNAcylation homeostasis in NP tissue may be a promising novel therapeutic target for the prevention and treatment of IDD.

## Supplementary information

Supplementary information
