# Supplementary material for: O-GlcNAc transferase regulates intervertebral disc degeneration by targeting FAM134B-mediated ER-phagy
Source: Exp Mol Med. 2022 Sep 2;54(9):1472–85. doi: 10.1038/s12276-022-00844-7 (PMC9535016; doi:10.1038/s12276-022-00844-7)
Supplement: Supplementary file 1 — Supplementary information [file 12276_2022_844_MOESM1_ESM.pdf]

Supplementary Information

Materials and methods

Cell viability

Cell viability was assessed using the Cell Counting Kit-8 assay (CCK-8, Beyotime) according to the manufacturer's instructions. First, 1×10^4 cells/well were seeded in 96-well plates and exposed to ND for different times. Thereafter, 10 μL of CCK-8 reaction solution was added to each well, cultured at 37 °C for 2 h, and the absorbance value was measured at 450 nm using a spectrophotometer (BioTek, Winooski, VT).

Western blot analysis

The corresponding protein extraction kits (Beyotime, Nantong, China) were used to lyse cells and extract protein samples according to the kit instructions. 40 μg protein of each sample were loaded onto appropriate 8%–12% sodium dodecyl sulfate-polyacrylamide gels to separate protein bands based on the molecular weight of the target protein and transferred to polyvinylidene difluoride membranes (Merck Millipore, Darmstadt, Germany). Then, the membranes were incubated with primary antibodies at the appropriate dilutions overnight at 4 °C prior to incubation with horseradish peroxidase-conjugated secondary antibodies for 2 h at room temperature, detection with an enhanced chemiluminescence imaging system (Bio-Rad) and quantification of band densities with ImageJ software. The antibody dilutions were as follows: anti-OGT (1:1000), anti-O-GlcNAc (1:1000), anti-p16 (1:800), anti-p21 (1:2000), anti-p53

(1:1000), anti-Bcl-2 (1:1000), anti-Bax (1:2000), anti-cleaved caspase 3 (1:1000), anti-FAM134B (1:1000), anti-LC3B (1:600), anti-p62 (1:1000), anti-ubiquitin (1:1000), and anti-beta actin (1:2000). The dilutions of the secondary antibodies were as follows: goat anti-mouse IgG (H+L)-HRP (1:2000), goat anti-rabbit IgG (H+L)-HRP (1:2000), and mouse anti-rabbit IgG light chain-HRP (1:5000).

Immunofluorescence staining

Human NP cells seeded on coverslips were fixed with 4% paraformaldehyde for 20 min at room temperature, washed three times with PBS, 0.3% Triton X-100 was used to permeabilize for 15 min. Next, 10% goat serum was applied to block nonspecific binding for 30 min. The cells were incubated overnight at 4 °C with primary antibodies against OGT (1:100), O-GlcNAc (1:200), FAM134B (1:100), LC3B (1:100), p16 (1:100), and cleaved caspase 3 (1:100). Then, after washed three times with 0.1% PBST, the slides were incubated with CoraLite488- or CoraLite594-conjugated goat anti-rabbit/mouse secondary antibodies (1:100, Proteintech) for 1 h at room temperature. Fluorescence signals were visualized by fluorescence microscopy (Olympus, BX53, Melville, NY, USA) and analyzed using ImageJ software.

Apoptosis assay

An Annexin V-FITC/PI Apoptosis Detection Kit (KeyGEN, Nanjing, China) was used to evaluate apoptosis according to the manufacturer’s instructions. In brief, after the indicated treatment, NP cells were harvested with 0.25% trypsin (containing no EDTA).

After washed three times with PBS, the cells were stained with Annexin V-FITC (annexin V) and propidium iodide (PI) and analyzed using a flow cytometer (BD Biosciences, San Jose, CA, USA). the labeled cells of Annexin V+/PI- and Annexin V+/PI+ were considered as the apoptotic cells.

Transmission electron microscopy

Transmission electron microscopy (TEM) was used to detect the formation of autophagosomes and autolysosomes. In brief, the cells were collected and fixed with 2.5% glutaraldehyde for 4 h, rinsed in 0.1 M phosphate buffer (pH 7.4) and subsequently fixed with 1% osmium tetroxide (OsO_4) at room temperature for 2 h. After fixation, the samples were dehydrated twice through an ethanol gradient (30%-50%-70%-80%-85%-90%-95%-100%) and embedded in epoxy resin. After ultrathin (70 nm) sectioning, the sections were double stained with 2% uranyl acetate and lead citrate for 20 min at room temperature and examined using transmission electron microscopy (Tecnai, FEI, USA).

Coimmunoprecipitation

Cell samples were lysed with NP-40 lysis buffer (1% NP-40, 30 mM Tris-HCl (pH 7.4), 10 μg/mL aprotinin, 150 mM NaCl, 1 mM phenylmethylsulfonyl fluoride, and 10 μg/mL leupeptin) containing protease inhibitor cocktail (MCE) for 20 min on ice, the lysates were then centrifuged at 12,000 × g at 4 °C for 10 min, the supernatants were collected. Then, a bicinchoninic acid (BCA) kit (Beyotime) was used to measure the

total protein concentrations. After preclearing with 20 μL of protein A/G magnetic beads for 1 h at 4 °C, equal amounts of supernatants were incubated with IgG, anti-OGT, and anti-FAM134B antibodies plus protein A/G or anti-HA magnetic beads and anti-Flag magnetic bead conjugates overnight at 4 °C. Then, the beads were washed three times with NP-40 lysis buffer and boiled in 2 × SDS sample loading buffer for subsequent western blot analysis.

Supplemental Figures:

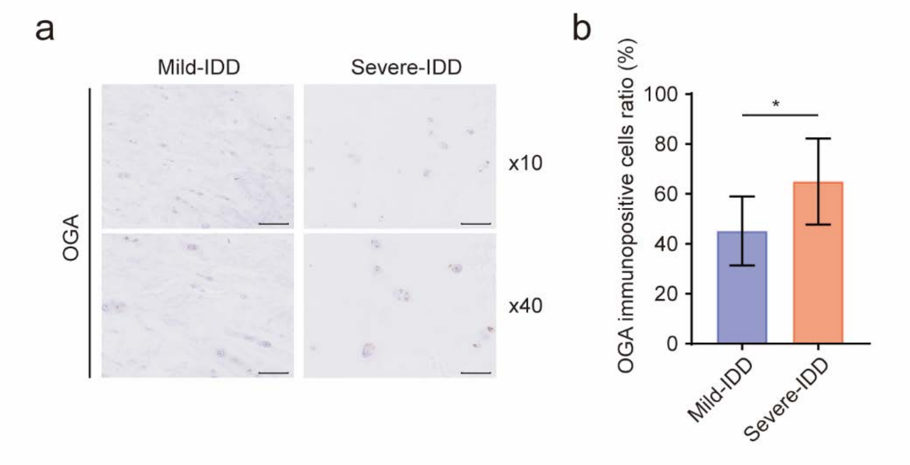

Supplementary Fig. 1 The protein expression profile of OGA in human NP tissues.

The protein expression level of OGA in human NP tissues was detected using IHC staining, representative images were shown (a) and relative OGA immunopositve staining cellratio were calculated (b), scale bar: 250 μm and 100 μm. The data are presented as the mean ± S.D. values. *P < 0.05.

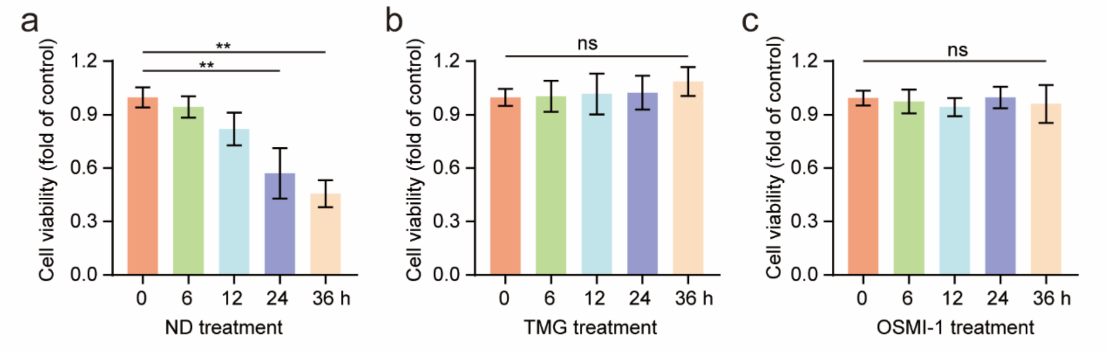

Supplementary Fig. 2 Effect of ND treatment, TMG, and OSMI-1 on cell viability in human NP cells. Atter exposed to ND treatment (a), TMG (b), and OSMI-1 (c) for the indicated time points (0, 6, 12, 24, and 36 hours), the viability of the human NP cells was measured by CCK-8 assay. The data are presented as the mean ± S.D. values. **P < 0.01, *P < 0.05.

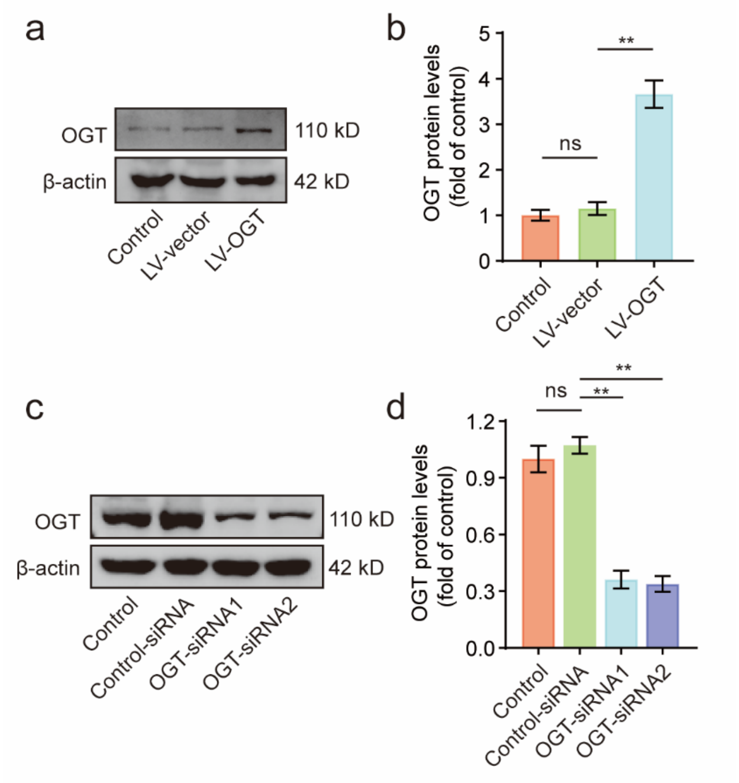

Supplementary Fig.3 Validation of OGT overexpression and knockdown in human NP cells.

(a, b) 72 hours post lentiviral transfection, the human NP cells were harvested and OGT expression were analyzed using western blot analysis, and the relative band densities were quantified. (c, d) 48 hours post siRNA transfection, the human NP cells were harvested and OGT expression were analyzed using western blot analysis, and the relative band densities were quantified. The data are presented as the mean ± S.D. values. **P < 0.01, *P < 0.05.

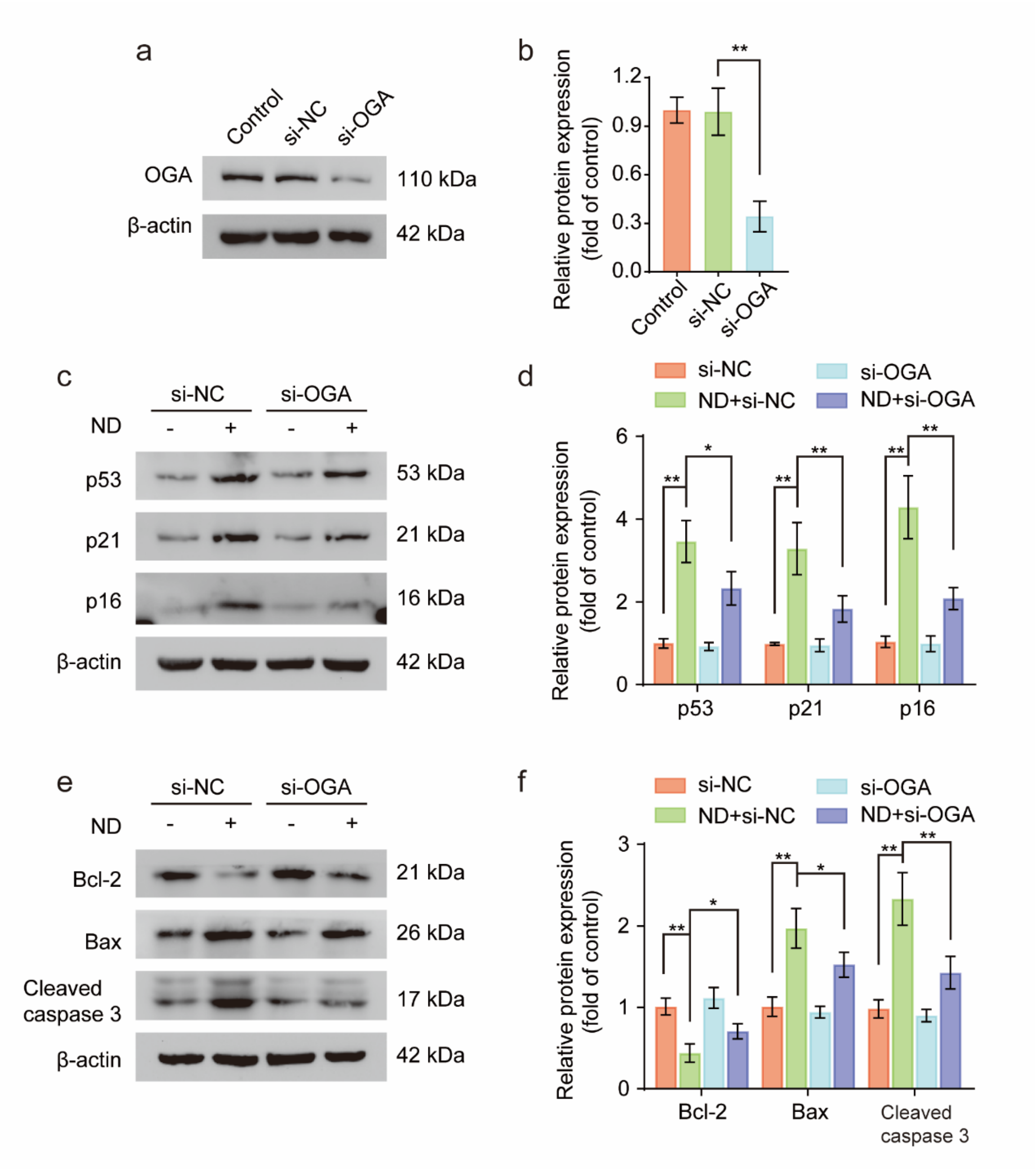

Supplementary Fig. 4 Effects of OGA knockdown on ND-induced senescence and apoptosis in human NP cells. (a, b) after transfected with OGA siRNA for 48 hours, the OGA knockdown efficacy in NP cells was validated by western blot analysis, and the relative band densities were quantified. (c-f) NP cells were transduced with the OGA siRNA for 48 h and then subjected to ND treatment for 36 h. The senescence- and apoptosis-associated proteins p53, p21, p16, Bcl-2, Bax, and cleaved caspase 3 were evaluated using western blot analysis, and the relative band densities were quantified. The data are presented as the mean ± S.D. values. $**P < 0.01$, $*P < 0.05$.

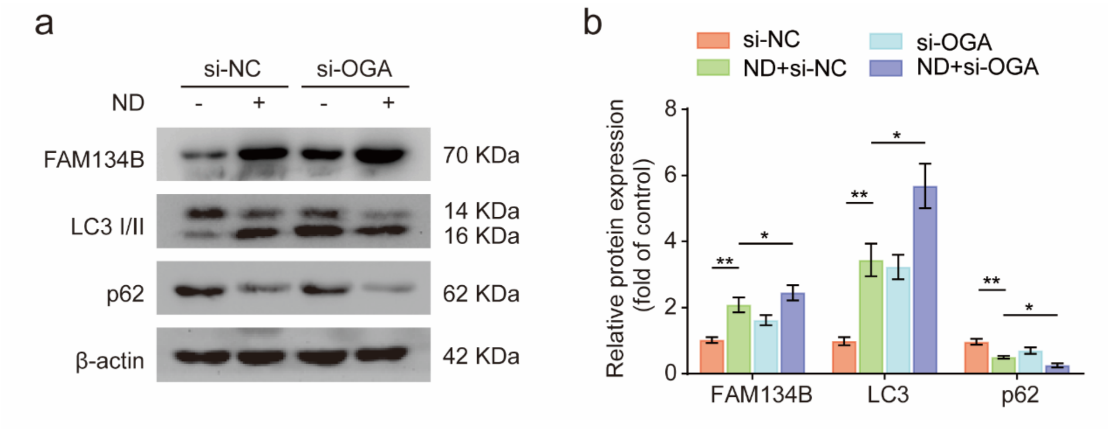

Supplementary Fig. 5 Effects of OGA knockdown on ND-induced FAM134B-mediated ER-phagy activation in human NP cells. (a, b) NP cells were transduced with the OGA siRNA for 48 h and then subjected to ND treatment for 36 h. ER-phagy associated proteins FAM134B, LC3, and p62 were assessed with western blot analysis, and the relative band densities were quantified. The data are presented as the mean ± S.D. values. $**P < 0.01$, $*P < 0.05$.

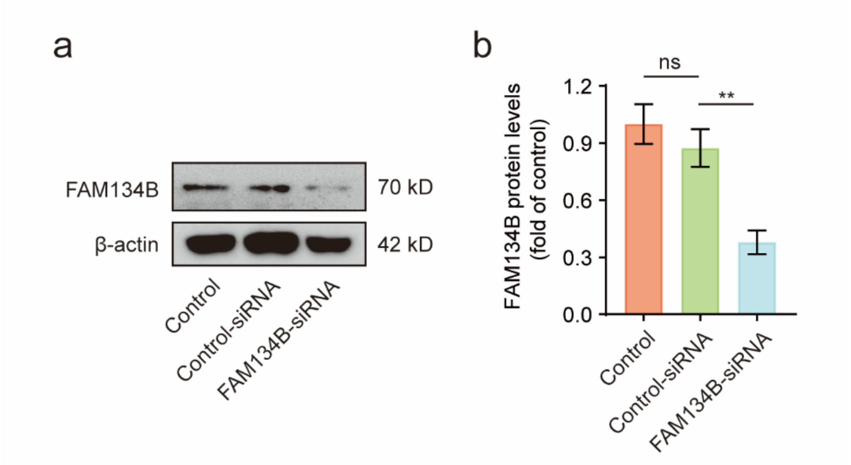

Supplementary Fig. 6 Validation of FAM134B knockdown in human NP cells.

(a, b) 48 hours post siRNA transfection, the human NP cells were harvested and FAM134B expression were analyzed using western blot analysis, and the relative band densities were quantified. The data are presented as the mean ± S.D. values. $**P < 0.01$, $*P < 0.05$.

Supplementary Tables

Supplementary Table 1 Patient demographics

**Case number**	**Gender**	**Age(years)**	**Pfirrmann grade**	**Diagnosis**
Case 1	male	13	I	Adolescent idiopathic scoliosis
Case 2	female	11	I	Adolescent idiopathic scoliosis
Case 3	female	14	II	Adolescent idiopathic scoliosis
Case 4	male	17	II	Adolescent idiopathic scoliosis
Case 5	female	17	II	Adolescent idiopathic scoliosis
Case 6	male	21	II	Adolescent idiopathic scoliosis
Case 7	female	43	IV	Lumbar spinal stenosis
Case 8	female	52	IV	Lumbar spinal stenosis
Case 9	male	57	IV	Lumbar spinal stenosis
Case 10	female	61	V	Lumbar spinal stenosis
Case 11	male	63	V	Lumbar spinal stenosis
Case 12	male	58	IV	Lumbar spinal stenosis

Supplementary Table 2 Reagents and antibodies used in this study

**Product name**	**Manufacturer**	**Catalog No.**
DMEM/F12	Gibco, USA	11330032
FBS	Gibco, USA	16000-044
PBS	Gibco, USA	70011-044
glucose-free DMEM	Gibco, USA	11966-025
thiamet G	MedChemExpress, USA	HY-12588
protein A/G magnetic beads	MedChemExpress, USA	HY-K0202
anti-HA magnetic beads	MedChemExpress, USA	HY-K0201
anti-Flag magnetic beads	MedChemExpress, USA	HY-K0207
OSMI-1	Abcam, USA	ab235455
Cycloheximide	Selleck, USA	S7418
ER-Tracker Green	Yeasen, China	40763ES20
LysoTracker Red	Yeasen, China	40739ES50
anti-OGT	Abcam, USA	ab96718
anti-p21	Abcam, USA	ab109520
anti-Bcl-2	Abcam, USA	ab196495
anti-Bax	Abcam, USA	ab104156
anti-LC3B	CST, USA	# 83506
anti-p16	CST, USA	#18769
anti-cleaved caspase 3	CST, USA	#9664
anti-FAM134B	Proteintech, China	21537-1-AP

anti-p62	Proteintech, China	18420-1-AP
anti-p53	Proteintech, China	10442-1-AP
anti-ubiquitin	Proteintech, China	10201-2-AP
anti-beta actin	Proteintech, China	66009-1-Ig
anti-O-GlcNAc	Novus, USA	10442-1-AP
goat anti-mouse IgG (H+L)-HRP antibody	Abcam, USA	ab6789
goat anti-rabbit IgG (H+L)-HRP antibody	Abcam, USA	ab6721
mouse anti-rabbit IgG chain-HRP	Abcam, USA	ab99697
goat anti-rabbit CoraLite488 antibody	Proteintech, China	SA00013-2
goat anti-mouse CoraLite594 antibody	Proteintech, China	SA00013-3
goat anti-mouse CoraLite488 antibody	Proteintech, China	SA00013-1
goat anti-rabbit CoraLite594 antibody	Proteintech, China	SA00013-4
